# *Mitreola brachypoda* (Loganiaceae), a new species from south-eastern Yunnan, China

**DOI:** 10.3897/phytokeys.277.195011

**Published:** 2026-07-22

**Authors:** Wen-Wen Pan, Chi Xiong, Fu-Guan Deng, Jing Wei, Jia-Xin Fu, Hai-Qing Lei

**Affiliations:** 1 Lishui Vocational & Technical College, Lishui, 323000, Zhejiang, China Guangxi Key Laboratory of Plant Conservation and Restoration Ecology in Karst Terrain, Guangxi Institute of Botany, Guangxi Zhuang Autonomous Region and Chinese Academy of Sciences Guilin China; 2 Hubei Provincial Key Laboratory for Protection and Application of Special Plant Germplasm in Wuling Area of China, Key Laboratory of State Ethnic Affairs Commission for Biological Technology, College of Life Sciences, South-Central Minzu University, Wuhan, 430074, Hubei, China Gesneriad Conservation Center of China (GCCC), Guilin Botanical Garden, Guangxi Zhuang Autonomous Region and Chinese Academy of Sciences Guilin China; 3 Yunnan Xianghao Agricultural Technology Co., Ltd., Kunming, 650500, Yunnan, China College of Tourism and Landscape Architecture, Guilin University of Technology Guilin China; 4 College of Tourism and Landscape Architecture, Guilin University of Technology, Guilin, 541006, Guangxi, China Lishui Vocational & Technical College Lishui China; 5 Guangxi Key Laboratory of Plant Conservation and Restoration Ecology in Karst Terrain, Guangxi Institute of Botany, Guangxi Zhuang Autonomous Region and Chinese Academy of Sciences, Guilin, 541006, Guangxi, China Hubei Provincial Key Laboratory for Protection and Application of Special Plant Germplasm in Wuling Area of China, Key Laboratory of State Ethnic Affairs Commission for Biological Technology, College of Life Sciences, South-Central Minzu University Wuhan China; 6 Gesneriad Conservation Center of China (GCCC), Guilin Botanical Garden, Guangxi Zhuang Autonomous Region and Chinese Academy of Sciences, Guilin, 541006, Guangxi, China Yunnan Xianghao Agricultural Technology Co., Ltd. Kunming China

**Keywords:** Limestone karst, Maguan, *
Mitreola
*, new taxon, taxonomy

## Abstract

A new species of Loganiaceae, *Mitreola brachypoda* W.W.Pan, F.G.Deng & C.Xiong, from the karst regions of south-eastern Yunnan, China, is described and illustrated. The new species is morphologically similar to *M. bullata* Y.S.Chen & J.J.Liao, but can be easily distinguished by a combination of characteristics, including the texture and shape of leaf blades, the type of inflorescence and corolla tube, the length of petiole, peduncle, corolla tube and lobes, as well as the colour of lateral veins and corolla. At present, only one population of approximately 30–40 mature individuals has been confirmed from the type locality. The species is provisionally assessed as Data Deficient (DD) according to the IUCN Red List Categories and Criteria.

## Introduction

The genus *Mitreola* L., belonging to the family Loganiaceae, is a small genus comprising 22 species worldwide (Tran et al. 2025; POWO 2026), distributed in Africa, America, Asia, Oceania and the Pacific Islands (Leenhouts 1962, 1972; Leeuwenberg and Vidal 1972; Leeuwenberg 1974; Li and Leeuwenberg 1996; Islas-Hernández et al. 2019). Southern and south-western China represent the primary centre of diversity for *Mitreola*, with 15 species documented in the country, 13 of which are endemic; the majority of these species are restricted to limestone karst habitats (Li 1979; Fang et al. 1995; Li and Leeuwenberg 1996; Ma et al. 2010; Shan et al. 2019, 2021; You et al. 2020; Liao and Chen 2021; Liu et al. 2022, 2023; Hu et al. 2023).

In March 2026, during a survey in the karst regions of Maguan County, southern Yunnan, we discovered an unknown species of *Mitreola*, characterised by obovate to spatulate leaves and a campanulate, pale purple corolla. Detailed morphological analysis, including *in situ* observations, measurements and floral dissections, was conducted and voucher specimens were collected. After careful comparison with known *Mitreola* species from China and adjacent regions, along with a comprehensive review of herbarium specimens and published literature (Fang et al. 1995; Li and Leeuwenberg 1996; Ma et al. 2010; Shan et al. 2019, 2021; You et al. 2020; Liao and Chen 2021; Liu et al. 2022, 2023; Hu et al. 2023; Nuraliev et al. 2023; Tran et al. 2025), we confirmed that the newly-discovered material represents a new species of *Mitreola*.

## Materials and methods

Plant photographs were obtained *in situ* using a smartphone. Morphological observations of herbarium specimens and dissections were made using a Nikon D7200 (Japan, Nikon Co., Ltd.). Descriptive terminology follows Beentje (2016). Voucher specimens prepared from the field-collected material were deposited at the Herbarium of Guangxi Institute of Botany (**IBK**). Herbarium acronyms follow Index Herbariorum (Thiers 2026). For comparative studies, digital images of additional specimens of closely-related species from Herbaria (BNU, E, GXMI, IBK, IBSC, K, KUN, P, PE etc.) were examined via the Chinese Virtual Herbarium (https://www.cvh.ac.cn/) and the Global Biodiversity Information Facility (https://www.gbif.org/).

## Taxonomic treatment

### Mitreola brachypoda

Taxon classificationPlantaeGentianalesLoganiaceae

W.W.Pan, F.G.Deng & C.Xiong sp. nov.

9176BF62-C5F0-53B5-9EBC-480010755821

urn:lsid:ipni.org:names:77387601-1

Figs 1, 2

#### Type.

China • Yunnan Province: Wenshan Zhuang and Miao Autonomous Prefecture, Maguan County, Gulinqing Township, Kashang Village; 22.80°N, 104.05°E; elev. ca. 1450 m, 8 March 2026; *Wen-Wen Pan et al. PWW260308-01* (fl.) (holotype IBK!, IBK00473504; isotypes IBK!, IBK00473505, 00473506).

**Figure 1.**
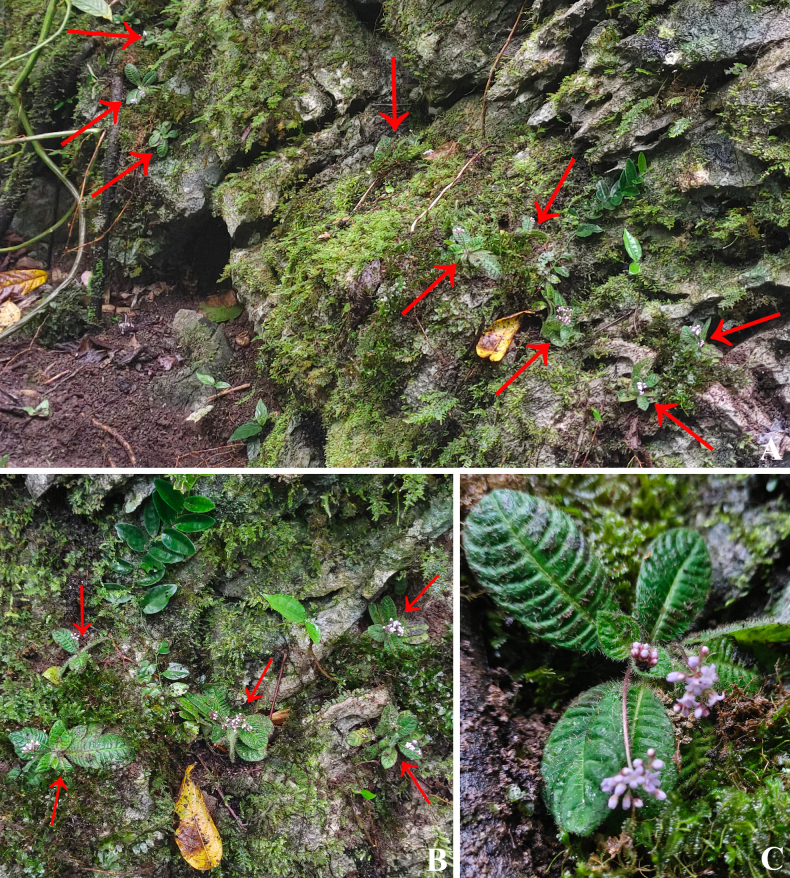
Habitat and habit of *Mitreola brachypoda* W.W.Pan, F.G.Deng & C.Xiong, sp. nov. **A, B**. Habitat, arrows indicate plants; **C**. Plant habit (all photographed by Fu-Guan Deng).

**Figure 2.**
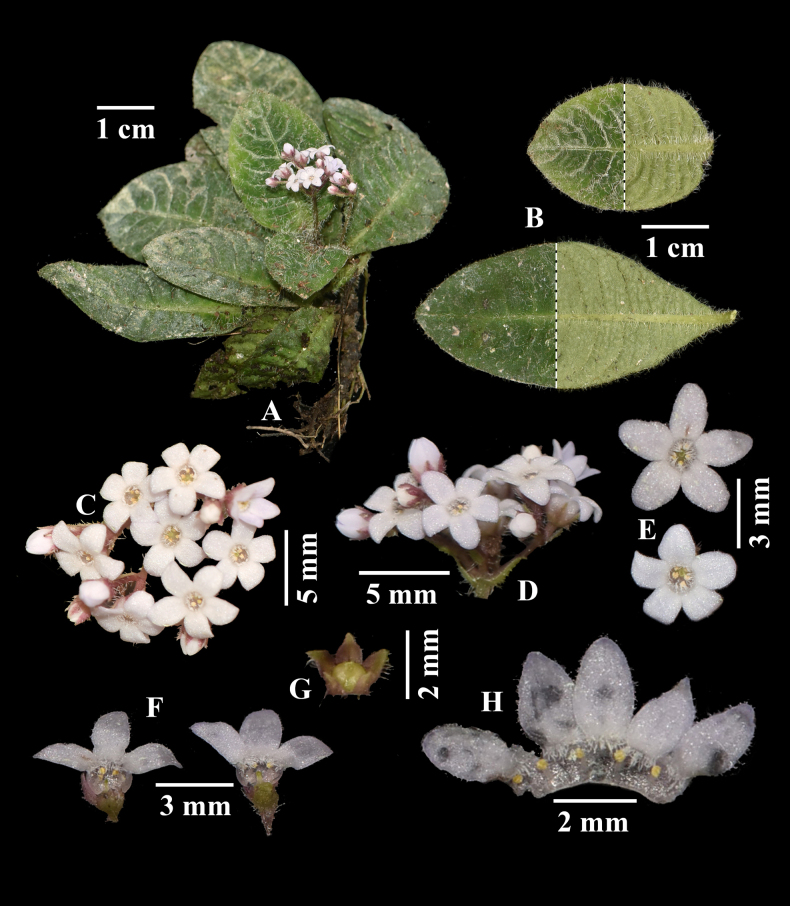
*Mitreola brachypoda* W.W.Pan, F.G.Deng & C.Xiong, sp. nov. **A**. Flowering plant; **B**. Adaxial (left) and abaxial (right) surfaces of leaf blade; **C**. Top view of inflorescence; **D**. Side view of inflorescence; **E**. Front view of flower; **F**. Longitudinally dissected flower, lateral view; **G**. Pistil and sepals; **H**. Opened corolla showing stamens (all photographed by Jing Wei).

#### Diagnosis.

*Mitreola brachypoda* resembles *M. bullata*, but differs by its 0–5 mm long petiole (vs. 10–26 (–35) mm), its obovate to spatulate leaf blade with cuneate to attenuate base and acute, obtuse to rounded apex (vs. elliptic or long elliptic, base cuneate or rounded, apex acute or obtuse), its dense umbel-like thyrsoid inflorescence with several cymes (vs. dichasium, sparse), its white or pale purple campanulate corolla (vs. urceolate, light purple), its ca. 1 mm long tube (vs. ca. 1.5 mm) and its 2.3–2.5 mm long lobes (vs. 1.3–1.5 mm) cacacaca.

#### Description.

Perennial lithophytic herbs up to 15 cm high. **Stem** proximally prostrate, terminally erect to suberect, terete, 2–5 cm long, ca. 3 mm in diam., simple, sparsely pilose when young, glabrescent with age, pale green; **internodes** 2–8 mm long. **Leaves** opposite, often clustered at the stem or branch apex, petiolate; **petiole** short, 0–5 mm long, ca. 1.5 mm in diam., light green, sparsely pilose; **leaf blade** papery when dry, obovate to spatulate, 1.8–5.5 × 1.5–2.6 cm, adaxially dark green to green, abaxially light green, sparsely pilose on both surfaces, especially on the veins; base cuneate to attenuate; margin entire, ciliate; apex acute, obtuse to rounded; lateral veins 5–9 (–12) on each side of mid-rib, adaxially sunken, abaxially protuberant; **stipules** triangular, ca. 1 mm long, interpetiolar. **Inflorescences** terminal and axillary, usually 2–4 per plant, umbel-like thyrsoids with several dense cymes, terminal and axillary, usually 2–4 per plant, 8–15 flowers per cyme; peduncle terete, 3–12 cm long, ca. 1 mm in diam., purplish-red, sparsely pilose; **bracts** lanceolate, 2–2.5 mm long, villous; pedicels terete, 1–1.5 mm long, ca. 0.5 mm in diam., purplish-red, sparsely pilose. **Flowers** actinomorphic, pentamerous. **Calyx** lobes 5, ovate, 1.5–1.8 × 0.6–0.8 mm, purplish-red, sparsely pilose along the keel on the outside, margin entire, apex acute. **Corolla** campanulate, white or pale purple, glabrous outside, ca. 5 mm in diam.; tube ca. 1 mm long, with a ring of translucent hairs at throat, hairs ca. 0.5 mm long, spreading horizontally; lobes 5, ovate, 2.3–2.5 × 1.5–1.8 mm, margin entire, ciliate, apex rounded. **Stamens** 5, inserted near middle of corolla tube, glabrous; filaments ca. 0.3 mm long; anthers ovate, ca. 0.2 mm long. **Ovary** semi-inferior, bilocular, ca. 0.5 × 1.0 mm; **style** ca. 0.5 mm long, free at base, stigma capitate. **Capsules** not seen.

#### Phenology.

Flowering from February to April.

#### Etymology.

The specific epithet ‘*brachypoda*’ is derived from the Greek ‘*brachys*’ (short) and ‘*podos*’ (stalk), in reference to its distinctly short petioles.

#### Vernacular name.

duǎn bǐng dù liáng cǎo (Chinese pronunciation); 短柄度量草 (Chinese name).

#### Distribution and ecology.

This new species is currently found only at the type locality at an elev. of ca. 1450 m. It grows on moist, moss-covered cliffs (Fig. 1). Associated vascular species include *Lepisorus fortunei* (T.Moore) C.M.Kuo (Polypodiaceae) and *Piper sintenense* Hatus. (Piperaceae).

#### Conservation status.

Based on current field investigations, the new species is known only from its type locality, with approximately 30–40 mature individuals observed. The data currently available are insufficient to comprehensively assess its conservation status. According to the IUCN Red List Categories and Criteria (IUCN 2026), it is provisionally assessed as Data Deficient (DD). Further botanical surveys in adjacent areas and long-term monitoring are required to better evaluate its distribution, population size and potential threats.

## Discussion

*Mitreola brachypoda* is morphologically most similar to *M. bullata*, but can be reliably distinguished by a combination of vegetative and reproductive traits, including short petioles (0–5 mm), obovate to spatulate leaf blades, a dense umbel-like thyrsoid inflorescence, a campanulate corolla with a short tube (ca. 1 mm long, tube length half of or less than the lobes), while in *M. bullata*, the corolla tube is subequal to the lobes. These consistent differences support its recognition as a distinct species. While the shared characters of a semi-inferior ovary, capitate stigma and opposite leaves place it firmly within *Mitreola*, the short petioles and congested inflorescence suggest possible affinity with other karst-endemic species in southern China and northern Vietnam. The differences between *M. bullata* and the new species are provided in Table 1.

**Table 1.** Comparison of morphological characters of *Mitreola brachypoda* and *M. bullata*.

**Characters**	***M. brachypoda***	***M. bullata***
Petiole	0–5 mm long, light green, sparsely pilose	1.0–2.6 (–3.5) cm long, purplish-red or green, densely pilose
Leaf blade	obovate to spatulate, 1.8–5.5 × 1.5–2.6 cm; base cuneate to attenuate; apex acute, obtuse to rounded	elliptic or long elliptic, 1.8–5.2 (–8.5) × 0.8–1.7 (–3.2) cm; base cuneate or rounded; apex acute or obtuse
Leaf surface	not bullate, sparsely pilose on both surfaces, especially on the veins	bullate, densely pilose on both surfaces
Lateral veins	5–9 (–12) pairs, veins on abaxial surface light green	7–10 pairs, veins on abaxial surface purplish-red
Inflorescence	umbel-like thyrsoid with several cymes, dense	dichasium, sparse
Peduncle	3–12 cm long	up to 4.0 cm long
Corolla	campanulate, white or pale purple; tube ca. 1 mm long, lobes ovate, 2.3–2.5 mm long, margin ciliate	urceolate, light purple; tube ca. 1.5 mm long; lobes ovate, 1.3–1.5 mm long, margin glabrous

The discovery of *M. brachypoda* adds to the growing list of *Mitreola* species described from the karst landscapes of southern and south-western China, particularly from Yunnan Province. With 15 species currently recognised in China, 13 of which are endemic, the region — especially south-eastern Yunnan — stands out as a primary centre of diversity and endemism for the genus. Recent descriptions of *Mitreola crystallina* Y.M.Shui & W.H.Chen (You et al. 2020), *Mitreola bullata* Y.S.Chen & J.J.Liao (Liao and Chen 2021), *Mitreola lincangensis* Z.J.Mu, Z.J.Shan & B.Pan (Shan et al. 2021), *Mitreola viridiflora* C.Liu & S.W.Guo (Liu et al. 2023) and now *M. brachypoda* from Yunnan, along with *Mitreola quanruii* L.Wu & R.C.Hu (Hu et al. 2023) from Guangxi and *Mitreola sinhoensis* T.P.A.Tran, K.S.Nguyen, V.T.Bui, B.H.Quang & L.Wu (Tran et al. 2025) from northern Vietnam, highlight the ongoing discovery of range-restricted species in the Sino-Vietnamese karst belt. This biogeographic pattern suggests that the actual species richness of *Mitreola* may still be underestimated and that the limestone karsts of the border region function as both refugia and speciation drivers. From a conservation perspective, *M. brachypoda* is currently known only from a single population of 30–40 mature individuals at the type locality. Following IUCN Criteria, a provisional assessment of Data Deficient (DD) is appropriate, as insufficient information exists on its distribution range, population dynamics and potential threats. Nevertheless, given the fragmented and vulnerable nature of karst habitats under increasing anthropogenic pressure (e.g. agriculture expansion, tourism development), urgent field surveys in adjacent areas of Maguan County and across the border into northern Vietnam are warranted to better assess its true conservation status.

## Supplementary Material

XML Treatment for Mitreola brachypoda

## Additional information

### Artificial Intelligence (AI) use

The authors accept full responsibility for the content of the manuscript, including the disclosure of any use of AI.

No AI tools were used in the preparation of this manuscript.

### Author ORCIDs

W.-W. Pan https://orcid.org/0009-0003-9387-4283

C. Xiong https://orcid.org/0000-0002-9082-2539

F.-G. Deng https://orcid.org/0009-0007-1805-0785

J. Wei https://orcid.org/0009-0004-7378-1044

J.-X. Fu https://orcid.org/0000-0003-0792-5383

H.-Q. Lei https://orcid.org/0000-0001-8700-5872

### Data availability

All of the data that support the findings of this study are available in the main text.
